# Autophagy and Reactive Oxygen Species Are Involved in Neutrophil Extracellular Traps Release Induced by *C. albicans* Morphotypes

**DOI:** 10.3389/fmicb.2016.00879

**Published:** 2016-06-08

**Authors:** Samyr Kenno, Stefano Perito, Paolo Mosci, Anna Vecchiarelli, Claudia Monari

**Affiliations:** ^1^Division of Hygiene and Medical Microbiology, Innsbruck Medical UniversityInnsbruck, Austria; ^2^Microbiology Section, Department of Experimental Medicine, University of PerugiaPerugia, Italy; ^3^Internal Medicine, Department of Veterinary Medicine, University of PerugiaPerugia, Italy

**Keywords:** NET, autophagy, ROS, *C. albicans*, neutrophils

## Abstract

Neutrophil extracellular traps (NETs) are a combination of DNA fibers and granular enzymes, such as elastase and myeloperoxidase. In this study, we demonstrate that *Candida albicans* hyphal (CAH) cells and yeast (CAY) cells induce differential amounts, kinetics and mechanisms of NET release. CAH cells induced larger quantities of NET compared to CAY cells and can stimulate rapid NET formation up to 4 h of incubation. CAY cells are, also, able to induce rapid NET formation, but this ability was lost at 4 h. Both reactive oxygen species (ROS) and autophagy are implicated in NET induced by CAH and CAY cells, but with a time-different participation of these two mechanisms. In particular, in the early phase (15 min) CAH cells stimulate NET via autophagy, but not via ROS, while CAY cells induce NET via both autophagy and ROS. At 4 h, only CAH cells stimulate NET formation using autophagy as well as ROS. Finally, we demonstrate that NET release, in response to CAH cells, involves NF-κB activation and is strongly implicated in hyphal destruction.

## Introduction

*Candida albicans* is both the most common fungal commensal and the most important fungal pathogen causing mucosal and disseminated candidiasis in at risk population, especially immunocompromised patients. Phagocytes, particularly neutrophils, are believed to be the most effective cell type for clearing *Candida* infection (van ’t Wout et al., 1988; Cheng et al., 2012). It has been reported that the success of antifungal treatment in transplant recipients is, also, dependent on neutrophil recovery (Safdar et al., 2007). To disseminate in the host, *C. albicans* has evolved many different virulence traits. Among them, the ability to switch from yeast to a filamentous form (hyphae), in response to external signals, is a dominant one. This transition is important for invasion and tissue damage (Gow, 2002; Whiteway and Oberholzer, 2004).

Neutrophils are able to kill *C. albicans*, yeast and hyphal cells. However, the mechanism of killing is considered to be quite different because yeast forms are ingested and killed, while the hyphal forms are too large to be engulfed and killed intracellularly. Neutrophil antimicrobial mechanisms are well studied and include phagocytosis, degranulation, production of reactive oxygen species (ROS), antimicrobial peptides secretion and extrusion of neutrophil extracellular traps (NETs; Brinkmann et al., 2004; Nathan, 2006; Chen and Junger, 2012).

Neutrophil extracellular traps are a combination of DNA fibers and granular enzymes (Brinkmann et al., 2004), such as elastase and myeloperoxidase, that are released, mainly, via the cell-death program named NETosis by Steinberg and Grinstein (2007). Activation of NETosis has been shown to involve NADPH oxidase (Nox2)-mediated oxidative burst (Babior, 1999), disintegration of the nuclear envelope and most granule membranes, which, together, result in massive vacuolization (Fuchs et al., 2007), intracellular de-condensation of nuclear chromatin (Wang et al., 2009), and eventually formation of NET (Brinkmann et al., 2004). NET formation is known to be stimulated by some cytokines (e.g., Interleukin-8), bacterial products (lipopolysaccharide, LPS) and, importantly, by clinically relevant bacteria such as *Shigella flexneri* (Brinkmann et al., 2004), *Staphylococcus aureus* (Brinkmann et al., 2004), *Streptococcus pneumoniae* (Brinkmann and Zychlinsky, 2007), and by fungi including *C. albicans* and *Aspergillus fumigatus* (Urban et al., 2006, 2009; Bruns et al., 2010; Branzk et al., 2014). Recently, rapid extrusion of NETs in response to microorganisms, such as *S. aureus* (Pilsczek et al., 2010) and *C. albicans* (Byrd et al., 2013) has been reported to occur without cell death.

It has been, recently, demonstrated that NET formation, could be secondary to superoxide production and autophagy (Remijsen et al., 2011). Autophagy is a well-conserved, essential intracellular degradation process, known to regulate protein and organelle turnover in many cells (Levine and Kroemer, 2008). In addition, autophagy has been implicated, also, in cell death (Kroemer et al., 2009). Recent data provide evidence for autophagy’s contribution to distinct antimicrobial strategies, rather than to neutrophil development or survival. An autophagy response is triggered, for example, by group A *Streptococcus* (Nakagawa et al., 2004), *Mycobacterium tuberculosis* (Gutierrez et al., 2004), *Salmonella enterica* (Birmingham and Brumell, 2006), *Toxoplasma gondii* (Ling et al., 2006), *Aspergillus niger* (Nitsche et al., 2013) *Cryptococcus neoformans* and *C. albicans* (Nicola et al., 2012).

Although it is demonstrated that *C. albicans* induces NET-release (Urban et al., 2006), the molecular mechanisms involved in this process have yet to be completely elucidated. Recently it has been demonstrated that *C. albicans* spores, both live and heat killed, induce autophagy (Kanayama and Shinohara, 2016), however, its role is not described in NET induction by different morphotypes of *C. albicans*. The aim of this study was to gain some insight into the mechanisms involved in the NETs formation, including the role of autophagy, and to differentiate the relevance of hyphal and yeast forms, of the fungus, during this process.

## Materials and Methods

### Reagents and Media

RPMI 1640 medium with L-glutamine, without phenol red and sodium bicarbonate were obtained from Sigma (St Louis, MO, USA). Rabbit polyclonal antibodies to neutrophil elastase (H-57) and to actin (H300) and goat antibody to rabbit IgG-TR conjugated were obtained from Santa Cruz Biotechnology (Dallas, TX, USA). Rabbit monoclonal antibody to human LC3B (D11), rabbit polyclonal antibodies to human pNF-κB p65 (Ser 536) and to human NF-κB p65 (C-20) were purchase from Cell Signaling Technology (Danvers, MA, USA). PE-conjugated rabbit monoclonal antibody (IgG) to human pNF-κBp65 (Ser 536) and rabbit monoclonal antibody isotype-matched control (PE-conjugated) were obtained from Cell Signaling Technology (Danvers, MA, USA). SYTOXgreen Nucleic Acid Stain was purchase from Invitrogen (Carlsbad, CA, USA). Human serum (HS) was obtained from MP biomedical (Santa Anna, CA, USA). Normal goat serum was obtained from Jackson Immuno Research (Newmarket, UK). Cold water fish gelatin, bovine serum albumin (BSA), Wortmannin (WT), *N*-acetyl-L-cysteine (NAC), Tween 20, 5-ammino-2,3-diidro-1,4-ftalazindione (luminol), water DNase free, phosphate buffered saline (PBS), DNAse-1 and Cytochalasin D (cytD) were purchase from Sigma–Aldrich (St Louis, MO, USA). The NF-κB inhibitor, (E)-3-[4-*t*-Butylphenylsulfonyl]-2-propenenitrile (BAY 11-7082), was obtained from Santa Cruz Biotechnology. Mammalian protein extraction reagent (M-PER) was purchased from Thermo Scientific (Rockford, IL, USA). Ficoll-Paque PREMIUM was obtained from General Healthcare (Buckinghamshire, UK).

### Isolation of Human Neutrophils

Heparinized venous blood from healthy donors was diluted with RPMI 1640, and mononuclear cells were separated by Ficoll-Paque density gradient centrifugation (Monari et al., 2002). The pellet containing neutrophils and erythrocytes was treated with hypotonic saline to lyse the erythrocytes. Granulocytes were collected by centrifugation, washed twice in RPMI 1640, counted, and adjusted to the desired concentration. The purity of neutrophils (PMNs) isolated by this method was always >98%, as determined by Giemsa staining. PMNs viability, evaluated after 18 h of incubation, was >98% in all determinations by a trypan blue dye exclusion test.

### Candida albicans

The strains of *C. albicans* used in this study were *C. albicans* virulent strain CA-6, isolated from clinical specimen (Vecchiarelli et al., 1988), and *C. albicans* (CA1398) carrying the *ACT1p-gLUC59* fusion (gLUC59). The *gLUC59* luciferase reporter has previously been described (Pietrella et al., 2012).

*Candida albicans* was cultured at 30°C in yeast extract-peptone-dextrose (YPD) broth overnight under slight agitation. Yeast cells, grown to stationary phase, were harvested from overnight YPD culture and resuspended to a final concentration of 5 × 10^7^ cells/ml in YPD to induce yeast form, or in RPMI-1640 2% glucose to induce hyphal form for 4 h at 37°C. The cells were washed twice, counted on a hemocytometer, and adjusted to the desired concentration. Hyphal form was counted six times to overcome the problem of hyphae clumping (Urban et al., 2006).

### Detection of Neutrophil Extracellular Traps (NETs)

Staining with the non-cell-permeable DNA dye SYTOXgreen was used to study the kinetics of NET formation (Kirchner et al., 2012). Human neutrophils (2 × 10^6^/ml) in NET Medium (RPMI-1640 modified without phenol red and sodium hydrogen, Hepes 10 Mm), were seeded to a cellstar 96-well plate and incubated, for 15 min and 4 h at 37°C and 5% CO_2_, alone or with pre-opsonized (Urban et al., 2006) *C. albicans* hyphae (CAH), live (CALY) or heat inactivated yeast (CAIY; E:T = 1:2). To detect the extracellular DNA of NETs, SYTOXgreen (2,5 μM; excitation: 488 nm, emission: 510 nm) was added to co-cultures and the fluorescence of NET-bound SYTOXgreen was analyzed using an infinite 200 reader (TECAN Infinite M200). In selected experiments, neutrophils, in NET Medium, were incubated alone or with CALY or CAIY at 37°C and 5% CO_2_. After 15 min of incubation the medium, of PMNs-CAIY co-cultures, was replaced with supernatants from PMNs-CALY co-cultures and NETs release was determined after 4 h of incubation as above described. To evaluate the ROS, autophagy and NF-κB role in NETs formation in response to *C. albicans*, PMNs, in NET Medium, were pre-treated with *N*-acetyl cysteine (NAC, 1 mM), a ROS scavenger, or WT (1 nM), an autophagy suppressor via inhibition of class III PI3K (Blommaart et al., 1997; Remijsen et al., 2011; Yang et al., 2013), or BAY 11-7082 (2.5 μM), an irreversible inhibitor of NF-κB activation, for 30 min at 37°C and 5% CO_2_. Then the cells were incubated alone or with pre-opsonized CAH (E:T = 1:2), CALY or CAIY (E:T = 1:2). Then NETs formation was determined as above described. Unstimulated cells were used as negative controls. WT and BAY 11-7082 were in DMSO and the final concentration did not have toxic effect (0,1% v/v).

### Microscopy Detection of NETs Formation

To visualize NETs, fluorescence microscopy was performed (Kirchner et al., 2012). Human neutrophils, 5 × 10^5^/500 μl in NET medium, settled in a chamber slide (Fisher Scientific, Rouchester, NY, USA), pre-treated or not with DNase-1 (100 U/ml; for 20 min at 37°C and 5%CO_2_), were incubated alone or with pre-opsonized CAH, CALY, or CAIY (E:T = 1:2), for 4 h at 37°C with 5% CO_2_. After incubation, the cells were fixed with 4% paraformaldehyde (PFA) for 10 min at room temperature. Subsequently, the supernatant was removed, and the air-dried chamber slides were rehydrated in PBS-DNase free and stained with SYTOXgreen (100 nM) for 60 min in the dark at room temperature. After washing three times with PBS-DNase free, the samples were mounted with Pro-Long and analyzed by fluorescent microscope (Leica DMRB).

For staining of neutrophil elastase, after rehydration, with PBS-DNase free, samples were blocked overnight at room temperature with 10% normal goat serum, 5% cold water fish gelatin,1% BSA and 0,05% tween-20 diluted in PBS-DNase free. Afterward, cells were washed with PBS-DNase free and incubated with antibody to neutrophil elastase rabbit (H-57; 1:50) for 60 min at 37°C. Then, the cells were washed three times with PBS-DNase free, incubated, in the dark, with TR conjugated goat antibody to rabbit IgG (1:100) for 60 min at 37°C, washed three times with PBS-DNase free and stained with SYTOXgreen (100 nM) for 60 min in the dark at room temperature. The microscopical analysis was performed as above described.

### Determination of ROS Production

Total ROS production was measured by chemiluminescent assay. Human neutrophils (4 × 10^5^/200 μl), pre-treated or not with NAC (1 mM), were incubated, in chemiluminescent medium (CL medium, RPMI-1640 modified without phenol red and sodium hydrogen, Hepes 20 mM), with CAH, CALY, or CAIY (E:T = 1:2), pre-opsonized with HS at 10% in RPMI for 30 min at 37°C and extensively washed after opsonization. 0.06 mM luminol was added to each sample and light emission was analyzed every 2 min for a period of 180 min by luminometer reader (TECAN Infinite M200). The light emission levels are expressed as RLU (relative light units = luminescence value given by the luminometer). As negative control cells were left in CL medium alone. Quantification of total ROS production was determined by calculation of area under curve (AUC; Ermert et al., 2009).

### Western Blotting for LC3B-II and pNF-κB

Human neutrophil (4 × 10^6^/200 μl) were incubated, in NET medium, with CAH, CALY, or CAIY (E:T = 1:2; pre-opsonized with HS at 10% in RPMI for 30 min at 37°C and extensively washed after opsonization) at 37°C with 5% CO_2_, for 15 min and 4 h. After incubation the cells were harvested, washed twice with PBS and the pellets were subjected to protein extraction with 20 μl of M-PER in presence of protease inhibitors (Sigma–Aldrich) and phosphatase inhibitors (Sigma–Aldrich). Protein concentrations were determined with a bicinchoninic acid (BCA) protein assay reagent kit (Pierce). The lysates (80 μg of each sample) were separated by sodium dodecyl sulfate 12% polyacrylamide gel electrophoresis (SDS-PAGE), and transferred to a nitrocellulose membrane (Pierce) for 90 min at 100 V in a blotting system (Bio-Rad) for Western Blot analysis. Membranes were placed in blocking buffer (3% no fat dry milk), and incubated overnight at 4°C with rabbit polyclonal antibodies to LC3B (D11; 1:1000), to pNF-κB p65 (Ser536; 1:200), or to NF-κB p65 (1:200). Immunoblotting with rabbit polyclonal antibody to actin (H300; 1:200) were used as internal loading controls to ensure equivalent amounts of protein in each lane. Detection was achieved using appropriate HRP-linked secondary antibodies, followed by Immun-Star^TM^ HRP chemiluminescent kit (Bio-Rad). Immunoreactive bands were visualized and quantified by Chemidoc Instruments (Bio-Rad).

### Flow-Cytometry Analysis of pNF-κB

Fresh human neutrophils (1 × 10^6^/500 μl) were incubated, in NET Medium, with CAH, CALY, or CAIY (E:T = 1:2; pre-opsonized with HS at 10% in RPMI for 30 min at 37°C), for 15 min and 4 h at 37°C and 5% CO_2_. Then the cells were collected, washed with PBS, fixed with 1.5% PFA for 10 min at room temperature, washed twice with PBS and permeabilized with Methanol for 10 min at 4°C. After permeabilization the cells were washed twice with PBS and incubated with rabbit monoclonal antibody to pNF-κB p65 (PE conjugated; 1:50) or with rabbit monoclonal antibody isotype-matched control (PE-conjugated; (1:50) for 30 min at 4°C. Cells were washed twice with PBS and 5000 events were analyzed by flow cytometry using FACS Calibur and Cell Quest Pro software (BD Biosciences, San Diego, CA, USA). Unstimulated neutrophils were used as control.

### Killing Experiments

Fresh human neutrophils were suspended in NET medium at concentration of 10^5^/100 μl in a 96 wells plate, pre-treated or not with DNase-1 (100 U/ml), to degrade extracellular DNA, or Cytochalasine D (cytD; 10 μg/ml), to block phagocytosis of fungi by neutrophils, for 20 min at 37°C and 5%CO_2_ (Urban et al., 2006), and incubated with *C. albicans* gLUC59 hyphae or *C. albicans* gLUC59 yeast, (pre-opsonized with HS at 10% in RPMI for 30 min at 37°C and extensively washed after opsonization), at an E:T = 10:1, at 37°C and 5%CO_2_ for 2 h (Enjalbert et al., 2009). Control cultures consisted of *C. albicans* gLUC59 hyphae or *C. albicans* gLUC59 yeast incubated without effector cells. After incubation 1 μl of coelenterazine (1 mg/ml in 1:10 methanol: H_2_O) was added to each well. The plate was briefly shaken and total bioluminescence emission was measured immediately and quantified with TECAN Infinite M200 using 1 s counting time. The light emission levels are expressed as RLU (relative light units = luminescence value given by the luminometer). Killing activity (Vecchiarelli et al., 1994; Enjalbert et al., 2009) vs. *C. albicans* was expressed as the percentage of RLU inhibition, according to the following formula: % killing activity = 100 – (RLU from the experimental group/RLU from control cultures) × 100. NET-mediated killing (Young et al., 2011) vs. *C. albicans* was determined by subtracting the extent of killing in the presence of DNAse-1 from total killing and expressed as the percentage of total killing reduction.

### Statistical Analysis

Data are reported as the mean ± standard error of the mean (SEM) from 3 to 7 separate experiments. Statistical analysis, was performed by using Student’s *t*-test. A value of *P* < 0.05 was considered significant.

## Results

### Induction of NETs Formation by *C. albicans* Morphotypes

To determine extracellular DNA released from human neutrophils (PMNs) stimulated with *C. albicans* morphotypes, PMNs were incubated with hyphal (CAH) or live yeast (CALY) cells for different time periods (15 min and 4 h) at 37°C and 5% CO_2_ and at an effector to target ratio 1:2, according to previous report (Ermert et al., 2009). In our experimental conditions *C. albicans* yeast does not undergo filamentation in 4 h of incubation, according with what previously demonstrated by Ermert et al. (2013).

**FIGURE 1:**
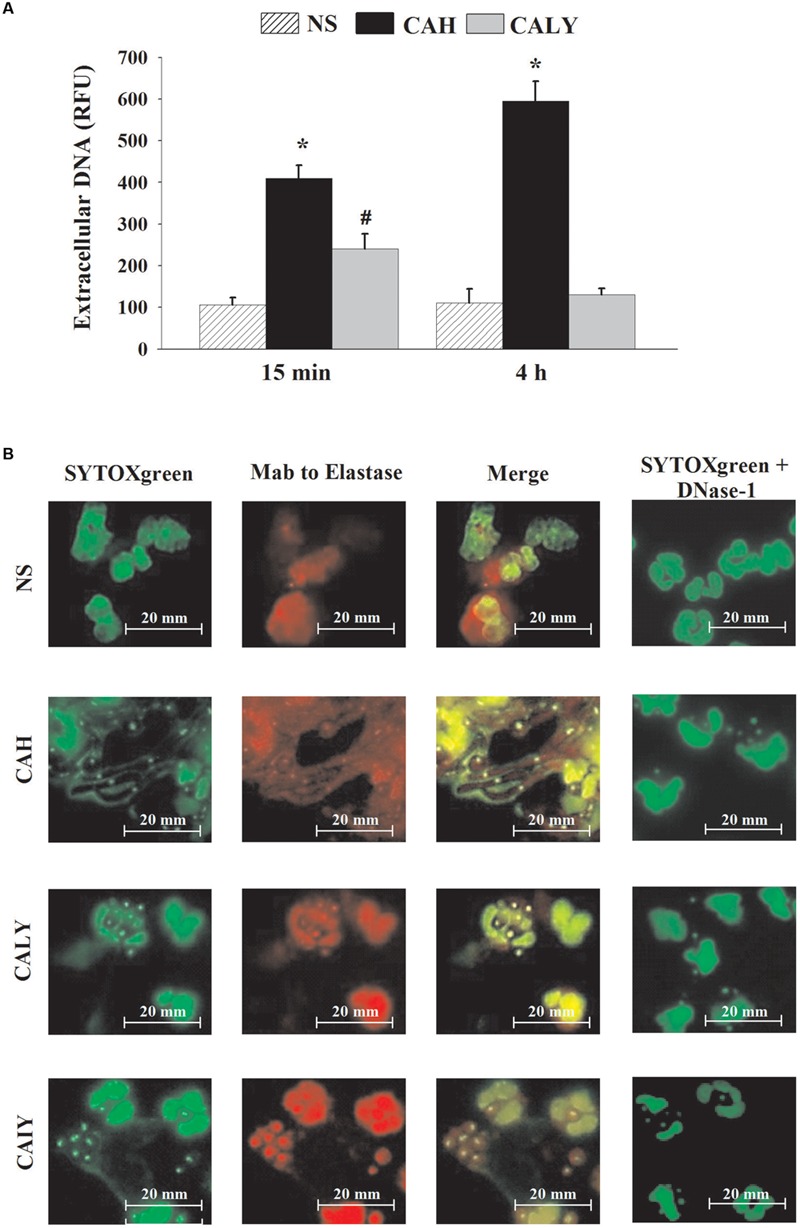
**NETs formation induced by *C. albicans*. (A)** Extracellular DNA released, in response to PMNs not stimulated (NS) or incubated with *C. albicans* hyphae (CAH) or live yeast (CALY), for 15 min or 4 h at 37°C and 5% of CO_2_, was determined using SYTOXgreen probe, as described in Section “Materials and Methods.” Bars are the mean ± SEM of *n* = 7 experiments with similar results. ^∗^*P* < 0.05 PMNs + CAH vs. PMNs NS; *^#^P* < 0.05 PMNs + CALY vs. PMN NS. **(B)** NET components (green: extracellular DNA; red: neutrophil elastase) were visualized by fluorescence microscopy after 4 h of PMNs incubation without stimuli (NS) or with *C. albicans* hyphae (CAH), live yeast (CALY), or heat inactivated yeast (CAIY). In the presence of DNAse-1 (100 U/ml), NET was completely degraded. Microphotography from a representative experiment (*n* = 3) are shown.

Extracellular DNA was quantified by using SYTOXgreen, a molecule that is fluorescent when it intercalates into strand of DNA, but does not enter live cells. The results (**Figure 1A**) show a significant early release of extracellular DNA from PMNs stimulated with both morphotypes. In particular, after 15 min of incubation, hyphal form induced significantly higher release of extracellular DNA in comparison to yeast form (*P* < 0.05)); after 4 h of incubation, CAH induced a progressive increase of extracellular DNA, whereas NET was negligible in response to CALY. For a better understanding the mechanism of time-dependent NET release, in response to live *C. albicans* yeast, we included, in our experimental system, heat inactivated yeast (CAIY).

To visualize NETs formation, neutrophils were co-incubated or not (NS) with CAH, CALY or CAIY for 4 h and stained with SYTOXgreen alone, with SYTOXgreen in the presence of mAb to Elastase, or DNase-1 (100 U/ml), that is able to degrade NET. As shown in **Figure 1B**, CAH induced large amounts of extracellular DNA release and neutrophils show delobulated nuclei. Conversely, the extracellular DNA was not observed after stimulation with CALY and neutrophils appear intact bearing lobulated nuclei. Surprisingly, the inactivated yeast-form (CAIY) is able to induce DNA release. To determine whether the low amount or the absence of extracellular DNA, released in response to live yeast, could be due to the metabolic activity of these cells, supernatant from co-culture of PMNs plus live *C. albicans* yeast (CALY) were harvested, after 15 min of incubation, and added to co-culture of PMNs plus heat inactivated *C. albicans* yeast (CAIY). After 4 h of incubation, extracellular DNA was quantified, by using SYTOXgreen; the results show that the addition of the supernatant caused a reduction of extracellular DNA release in response to CAIY of about 30%.

The capacity of NET induction was also tested by using heat inactivated hyphae and the results showed that they released NETs efficiently like live hyphae as previously published (Branzk et al., 2014).

### Inhibition of ROS Production Results in a Reduction of NETs Formation

**FIGURE 2:**
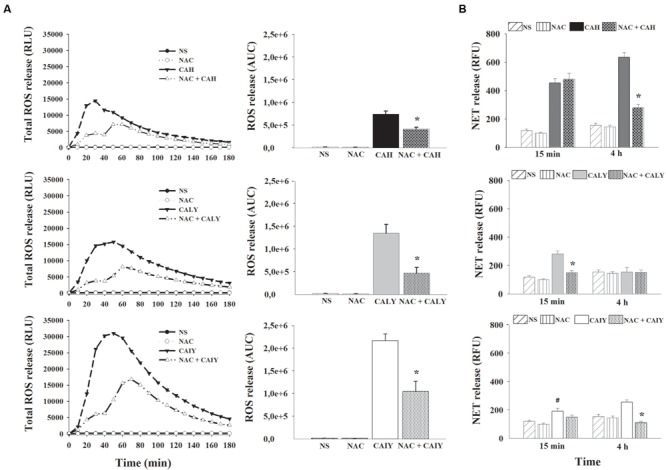
**Inhibition of ROS production causes a reduction of NETs formation *C. albicans* induced.** PMNs, pre-treated or not with N-acetyl L Cysteine (NAC, 1mM), were incubated without stimuli (NS) or with *C. albicans* hyphae (CAH), live yeast (CALY) or heat inactivated yeast (CAIY), and ROS production **(A)** and NETs formation **(B)** were determined as described in Section “Materials and Methods.” **(A)** The lines represent the kinetic of ROS release. The bars represent the total amounts of produced ROS obtained by analyzing the area under the curve (AUC). Lines are representatives of *n* = 5 experiments with similar results. Bars are the mean ± SEM of *n* = 5 experiments with similar results. ^∗^*P* < 0.05 PMNs NAC pre-treated vs. PMNs not pre-treated. **(B)** NET formation in response to CAH, to CALY or to CAIY after 15 min and 4 h of incubation. Bars are the mean ± SEM of *n* = 7 experiments with similar results. ^∗^*P* < 0.05 PMNs pre-treated with NAC + CAH or CALY vs. PMNs not pre-treated; *^#^P* < 0.05 PMNs + CAIY vs. PMNs NS.

Given that ROS production may participate in NET induction (Ermert et al., 2009), real time monitoring of total ROS was performed by using luminol enhanced chemiluminescence. The PMNs were pre-treated or not with *N*-acetyl-L-cysteine (NAC), a well-known antioxidant, and, then, CAH, CALY, or CAIY (E/T: 1/2) were added for 180 min. The results (**Figure 2A**) show that hyphae as well as, live or inactivated, yeast cells stimulated ROS production. The quantity of ROS released varied depending on the morphotype used. At any rate, ROS production, by each stimulator, was drastically reduced by NAC.

To determine if the inhibition of ROS production affected NETs release, in our experimental conditions, PMNs, pre-treated or not with NAC (1 mM), were incubated with CAH, CALY, or CAIY (E/T:1/2) for 15 min and 4 h at 37°C and 5% CO_2_. The results (**Figure 2B**) show that, after 15 min of incubation, NAC pre-treatment did not affect NET formation in response to CAH or CAIY, while caused significant inhibition in response to CALY. In addition, at this time, CAIY induced a significant early NETs release. By prolonging the incubation, until 4 h, a marked involvement of ROS in NET release was evidenced after stimulation with CAH and CAIY, as evidenced by significant NAC–mediated inhibition of NETs formation (**Figure 2B**).

### Inhibition of Autophagy Results in a Reduction of NETs Formation

It has been reported that autophagy pathway may be involved in NET induction (Remijsen et al., 2011), thus we evaluated whether, in our experimental conditions, the autophagy was induced by CAH, CALY, or CAIY. To this end the LC3B-II expression, marker of autophagy (Barth et al., 2010), was evaluated. PMNs, pre-treated or not with WT (1 nM), well known autophagy suppressor (Yang et al., 2013) via persistent inhibition of Class III PI3 kinases (Barth et al., 2010; Wu et al., 2010; Yang et al., 2013), were stimulated with CAH, CALY, or CAIY (E/T: 1/2), for 15 min and 4 h at 37°C and 5% CO_2_. Preliminary experiments showed that the optimal inhibition of LC3B-II expression was obtained at a dose of 1 nM of WT.

**FIGURE 3:**
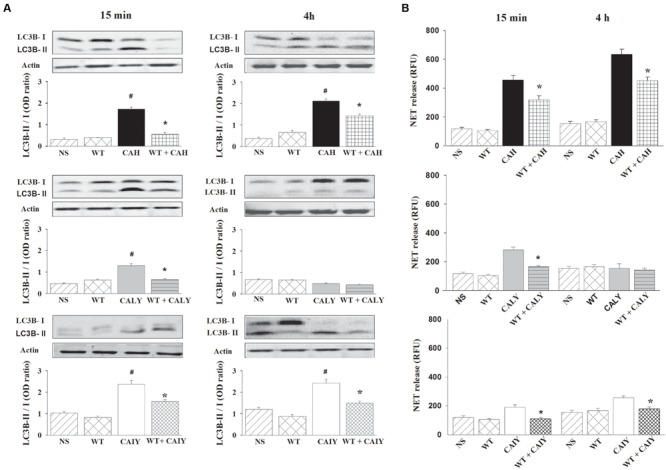
**Inhibition of autophagy causes a reduction of NETs release *C. albicans* mediated.** PMNs, pre-treated or not with WT (1 nM), were incubated without stimuli (NS) or with *C. albicans* hyphae (CAH), live yeast (CALY) or heat inactivated yeast (CAIY) and Western Blotting experiments for LC3B-II expression **(A)** and NET release determination **(B)** were performed as described in Section “Materials and Methods.” **(A)** LC3B-II expression in response to CAH, to CALY or to CAIY after 15 min and 4 h of incubation. Actin was used as loading control. Optical density of reactive bands was measured and LC3B-II/LC3B-I ratio was evaluated. PMNs treated with DMSO alone were also run in parallel and the results were similar to those obtained in absence of WT. Blots and bars are representative of experiments (*n* = 3) with similar results. ^#^*P* < 0.05 PMNs + CAH or CALY or CAIY vs. PMNs NS. **(B)** NETs formation in response to CAH, to CALY or to CAIY after 15 min and 4 h of incubation. PMNs treated with DMSO alone were also analyzed in parallel and the results were similar to those obtained in absence of WT. Bars are the mean ± SEM of *n* = 5 experiments with similar results.^∗^*P* < 0.05 PMNs pre-treated with WT + CAH or CALY or CAIY vs. PMN not pre-treated.

The results reported in **Figure 3A**, show a significant increase of LC3B-II expression after 15 min, in response to CAH, CALY as well as to CAIY and after 4 h in response to CAH and CAIY. The treatment with WT suppressed LC3B-II expression in all the cases in which activation was observed. We, next, examined the possible involvement of autophagy in NET release. To this end, PMNs, pre-treated or not with WT, were stimulated with CAH, CALY, or CAIY (E/T: 1/2) for 15 min and 4 h. The results (**Figure 3B**) show that WT pre-treatment produced a significant reduction of NETs formation in response to CAH, CALY, and CAIY after 15 min and in response to CAH or CAIY after 4 h.

### Inhibition of NF-κB Activation Results in a Reduction of NETs Release

**FIGURE 4:**
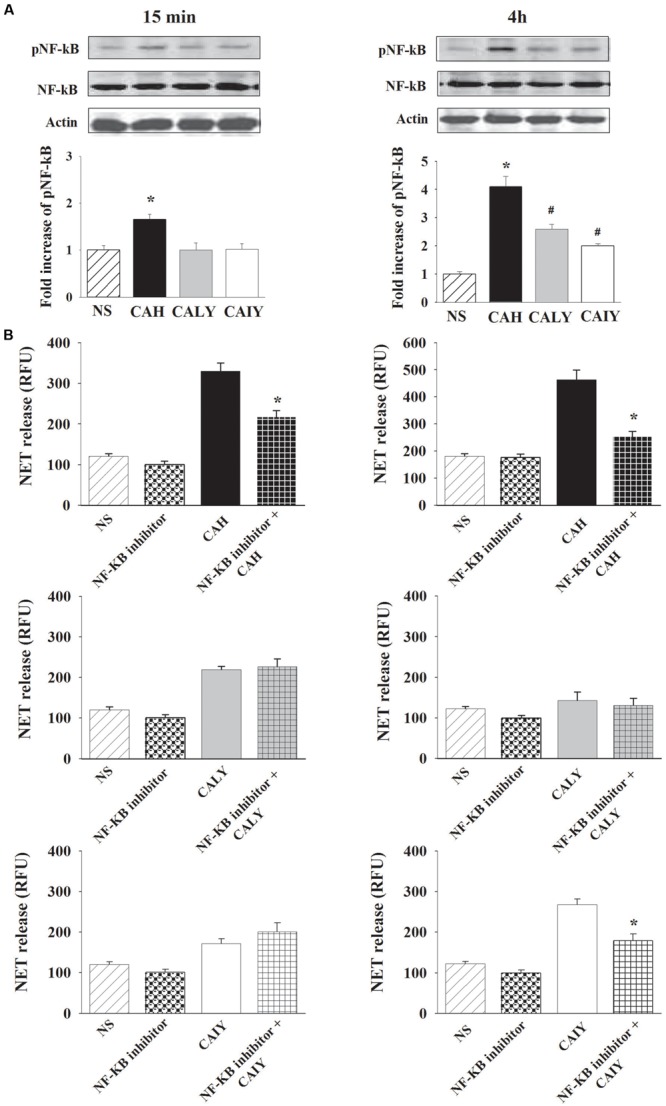
**Inhibition of NF-κB activation causes a reduction of NETs release *C. albicans* induced.** PMNs, were incubated without stimuli (NS) or with *C. albicans* hyphae (CAH), live yeast (CALY) or heat inactivated yeast (CAIY) and **(A)** Western Blotting analysis for pNF-κB and **(B)** NET release determination were performed as described in Section “Materials and Methods.” **(A)** NF-κB activation in response to CAH, to CALY or to CAIY after 15 min and 4 h of incubation. Optical density of reactive bands was measured and normalized by the NF-κB density in the same line. pNF-κB was quantified relative to PMNs NS. Actin was used as loading control. Blots are representative of experiments (*n* = 3) with similar results. Bars are the mean ± SEM of experiments (*n* = 3) with similar results. ^∗^*P* < 0.05 PMNs + CAH vs. PMN NS; *^#^P* < 0.05 PMNs + CALY or CAIY vs. PMN NS. **(B)** NET release determination in PMNs stimulated as above described, but pre-treated with NF-κB inhibitor (2.5 μM). The bars are the mean ± SEM of *n* = 5 experiments with similar results. ^∗^*P* < 0.05 PMNs pre-treated with NF-κB inhibitor + CAH or CAIY vs. PMNs stimulated with CAH or CAIY.

It has been reported that the activation of NF-κB p65 subunit is a relevant signaling pathway to the generation of DNA traps (Lapponi et al., 2013). Therefore, experiments were performed to analyze the possible involvement of NF-κB activation in NET release. First of all, we evaluated the activation of NF-κB in response to CAH, CALY, or CAIY. Western blotting analysis (**Figure 4A**) shows that *C. albicans* hyphae triggered a strong NF-κB activation after 15 min as well as after 4 h of incubation, while the *C. albicans* yeast, live and inactivated, stimulated NF-κB phosphorylation, only, after 4 h of co-culture with PMNs.

**FIGURE 5:**
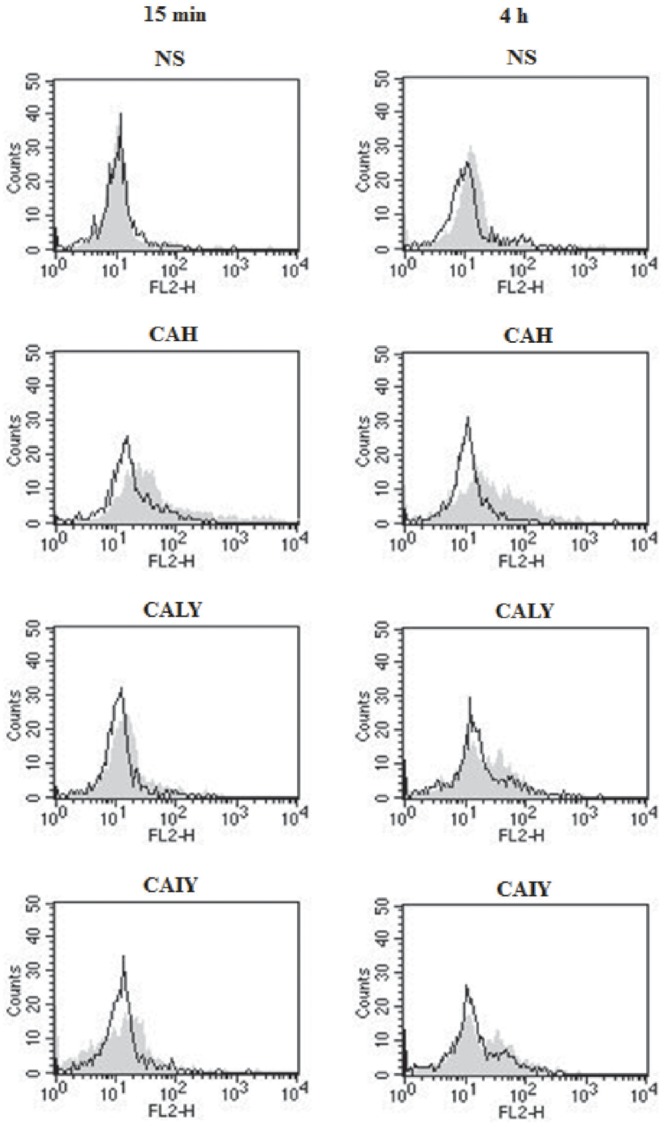
**Flow-cytometry analysis of pNF-κB.** PMNs, pre-treated or not with NF-κB inhibitor (2.5 μM), were incubated without stimuli (NS) or with *C. albicans* hyphae (CAH), live yeast (CALY) or heat inactivated yeast (CAIY) for 15 min and 4 h. Then cytofluorimetric analysis (events = 5000) was performed as described in Section “Materials and Methods.” Filled histograms represent fluorescence in absence of NF-κB inhibitor pre-treatment, black lines represent fluorescence in presence of NF-κB inhibitor pre-treatment. A PE-conjugated isotype-matched mAb was used as a negative control. Neutrophils untreated or treated with PE-conjugated isotype-matched mAb showed similar results. Histograms are representative of *n* = 3 experiments with similar results.

To assess a functional role for NF-κB in *C. albicans*-mediated NET release (**Figure 4B**), neutrophils were pre-treated or not with a NF-κB inhibitor (2.5 μM) for 30 min at 37°C (Lapponi et al., 2013) and, then, stimulated with CAH, CALY, or CAIY (E/T: 1/2) for 15 min and 4 h. The results show a significant inhibition of NET formation in response to hyphal form at both times. Regarding *C. albicans* yeast, pre-treatment with NF-κB inhibitor produced a down-regulation of NET release, only, in response to inactivated yeast in a 4 h of incubation. Preliminary experiments were performed using various doses of NF-κB inhibitor (BAY 11-7082; 1.25-2.5-5-10 μM). The results showed that the optimal dose to reduce NETs release in response to CAH or CAIY was 2.5 μM according to Lapponi et al. (2013), who consistently demonstrated that NF-κB inhibitor is able to inhibit both pNF-κB and NET induction. Indeed, by using cytofluorimetric analysis, we, also, demonstrated that the NF-κB inhibitor pre-treatment produced a down-regulation of pNF-κB expression in each case in which activation was observed (**Figure 5**).

### Role of ROS, Autophagy, and NF-κB in NETs Formation Induced by *C. albicans* Morphotypes

**FIGURE 6:**
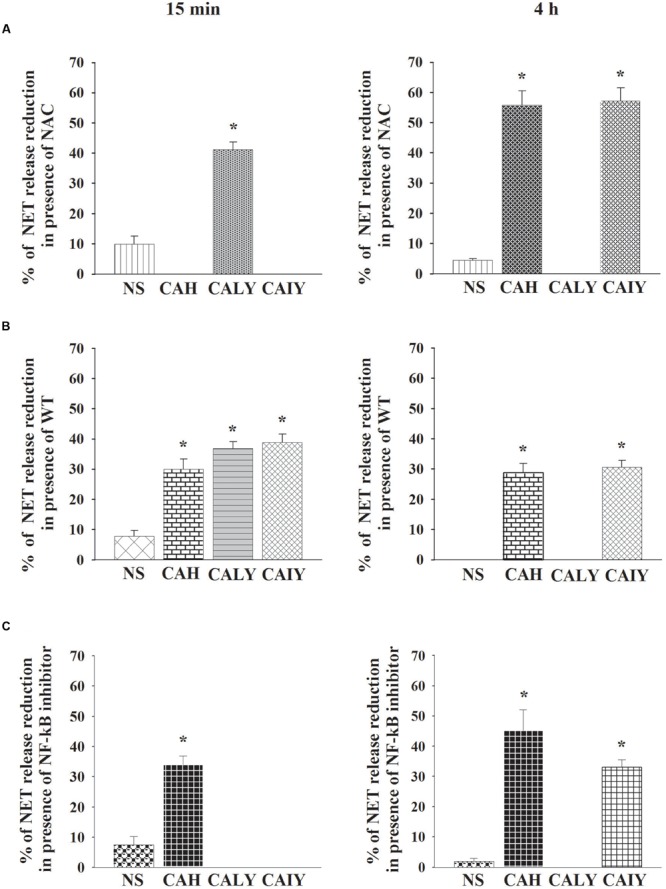
**Role of ROS production, autophagy and NF-κB in NETs released *C. albicans* induced.** PMNs, pre-treated with NAC **(A)**, or WT **(B)**, or NF-κB inhibitor (2.5 μM) **(C)**, were incubated without stimuli (NS) or with *C. albicans* hyphae (CAH), live yeast (CALY), or heat inactivated yeast (CAIY) for 15 min and 4 h. NETs formation was determined and percentage of NETs release reduction was calculated. The bars are the mean ± SEM of *n* = 5 experiments with similar results. ^∗^*P* < 0.05 PMNs + CAH or CALY or CAIY vs. PMNs without stimuli.

To better understand the role of ROS, autophagy, and NF-κB in NET generation, we determined the percentages of NETs reduction after treatment with NAC or WT or NF-κB inhibitor. The **Figure 6A** shows that, ROS production is involved, in the early NETs release (15 min) induced by live *C. albicans* yeast. Furthermore ROS is, also, involved in NETs released, by *C. albicans* hyphae and by inactivated *C. albicans* yeast in a 4 h of incubation.

The autophagy (**Figure 6B**), is implicated in rapid NET release stimulated by CAH, CALY, and CAIY and continues to be involved, until 4 h of incubation, in response to by CAH and CAIY. Regarding the role of NF-κB (**Figure 6C**) the results show that it is involved only in rapid NET formation stimulated by *C. albicans* hyphal form. By prolonging the incubation until 4 h NF-κB appears to be involved in NET induced by CAH and CAIY.

### NETs-Mediated Killing of *C. albicans* Hyphae

**FIGURE 7:**
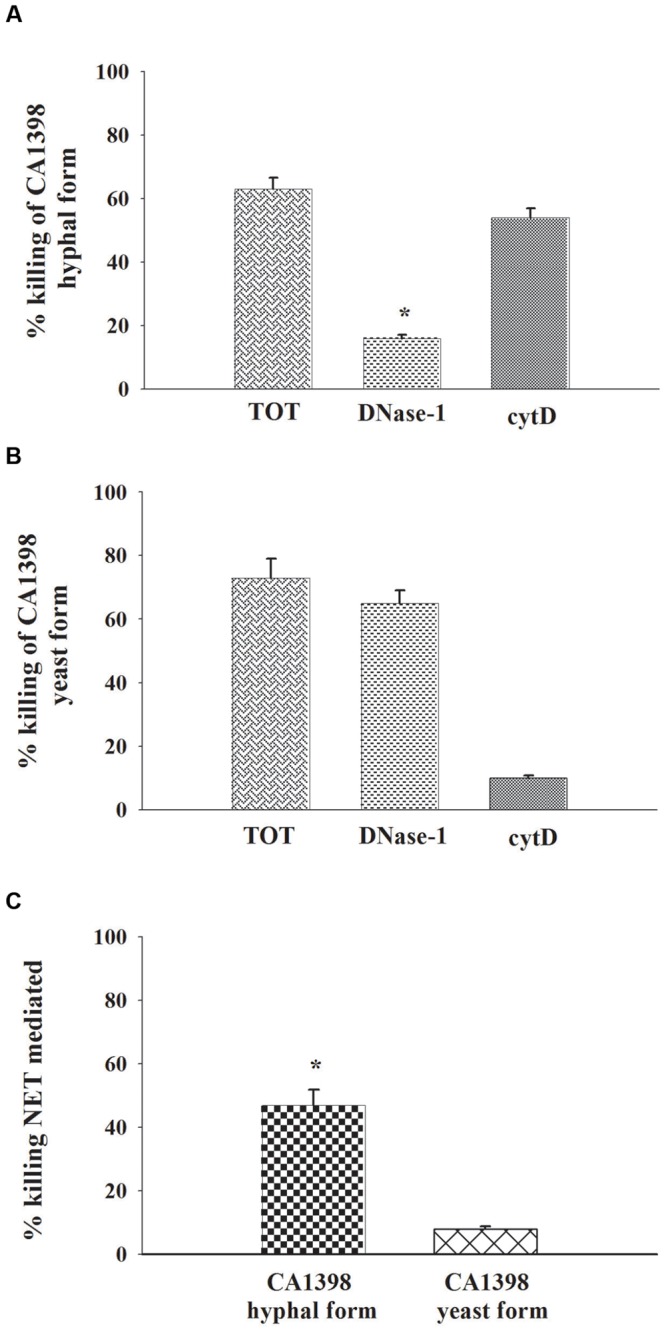
**NETs involvement on PMNs *C. albicans* killing.** PMNs killing activity vs. *C. albicans* hyphal and yeast forms. The PMNs, pre-treated, or not (total killing), with DNase-1 (100 U/ml; extracellular killing without NET-mediated killing), or with Cytochalasin D (cytD; 10 μg/ml; external killing), were incubated, for 2 h, with CA1398 hyphal **(A)** or yeast **(B)** forms as described in Section “Materials and Methods.” Bars are the mean ± SEM of experiments (*n* = 3) with similar results. ^∗^*P* < 0.05 CA1398 hyphal form killing by PMNs pre-treated with DNAse-I vs. PMNs untreated (TOT). **(C)** Percentage of killing NET mediated was calculated as described in Section “Materials and Methods.” Bars are the mean ± SEM of experiments (*n* = 3) with similar results.^∗^*P* < 0.05 CA1398 hyphal form vs. CA1398 yeast.

Finally the capacity of NETs to kill *C. albicans* hyphal and yeast forms, was explored. The killing was evaluated in the absence (total killing: intracellular + extracellular killing) or in the presence of DNase-1 (100 U/ml) to degrade extracellular DNA (to assess extracellular killing without NET-mediated killing), or of Cythocalasin D, (cyt D) to inhibit phagocytosis (to assess extracellular killing). The results show that *C. albicans* hyphal form was killed by PMNs, essentially by extracellular mechanisms (**Figure 7A**). Indeed DNase produced a strong inhibition of killing activity, while cytD did not affect killing. The analysis of live *C. albicans* yeast form killing by PMNs showed that yeast cells were quite entirely killed by intracellular mechanisms. Indeed DNAse did not inhibit the process, while the inhibition of phagocytosis resulted in almost complete inhibition of candidacidal activity (**Figure 7B**). In addition (**Figure 7C**) NET activity appears to be inconsistent in PMN killing of yeast form.

## Discussion

Neutrophils are immune cells that, freely, circulate in blood vessel and are considered to be crucials in the defense against infectious diseases. Invasive fungal infections, including candidiasis, represent a serious problem in neutropenic individuals (Horn et al., 2010), moreover the interaction of PMNs with *C. albicans* is a complex issue that has not been fully elucidated. While a positive role of PMN in systemic candidiasis is ascertained, a massive neutrophil recruitment is considered responsible of pathological inflammation during vaginal candidiasis (Van’t Wout et al., 1988).

Neutrophil extracellular traps is included among the mechanisms used by neutrophils to control microbial infections (Brinkmann et al., 2004), and it is able to degrade some microbial virulence factors and kill Gram-positive and Gram-negative bacteria (Matoszka et al., 2012).

In this study, we demonstrate that *C. albicans* hyphae and live yeast induce NETs release using autophagy and ROS with a different kinetics and participation of these two effector mechanisms. In addition, we here report that NETs release, in response to *C. albicans* hyphae, is regulated via NF-κB activation and is strongly involved in hyphal destruction.

Autophagy is a biological process of lysosome-mediated intracellular degradation enabling the routine turnover of proteins and organelles. It is considered a host cell effector mechanism to protect against pathogen invasion (Diacovich and Gorvel, 2010). Study by Casadevall’s group, showed that macrophage autophagy is important in immunity against *C. albicans* (Nicola et al., 2012). It is now known that NET formation could be dependent on autophagy for the unwinding and extrusion of DNA (Remijsen et al., 2011; Boone et al., 2015), however, the involvement of autophagy in NET formation during bacterial or fungal infection has not been elucidated. In this paper, we provide new evidence about the relationship between autophagy and NETs release in response to *C. albicans*. In particular autophagy plays an important role in triggering a rapid NETs induction in response to both morphotypes of *C. albicans*. Of note the involvement of autophagy was observed in all NETs inducing conditions in response to each morphotype. Moreover, the observation that autophagy, but not ROS, is involved in rapid NET release in response to hyphal form, is in agreement with a recent report (Byrd et al., 2013). Indeed, in this study, we demonstrate an early expression of LC3b, an autophagy marker, and this is consistent with recent data showing the presence of early and immature autophagic vacuoles in neutrophils stimulated with hyphae (Remijsen et al., 2011).

By prolonging the incubation time, both autophagy and ROS production appear to play a role in NETs release induced by hyphal cells. Overall, our findings reveal the implication of autophagy as a novel aspect of NETs release induced by *Candida* morphotypes.

Another novel observation from this study is that live *C. albicans* yeast cells are able to induce a rapid NET generation and that this process is dependent from both autophagy and ROS production.

Previous data (Branzk et al., 2014) showed the absence of NET formation in response to *C. albicans* yeast after 4 h of incubation. In this study, we confirmed the inability of *C. albicans* live yeast to induce NET in a 4 h of incubation, however, in a very early phase (within 15 min) NET formation was observed. Of note, differently from live yeast, inactivated yeast, was able to induce appreciable levels of NET release within 4 h. One possible explanation is that metabolic products released by live yeast, during the contact with neutrophils, could inhibit NET formation. This is supported by the observation that supernatant of live *C. albicans* co-cultured with neutrophils inhibits NET formation.

**FIGURE 8:**
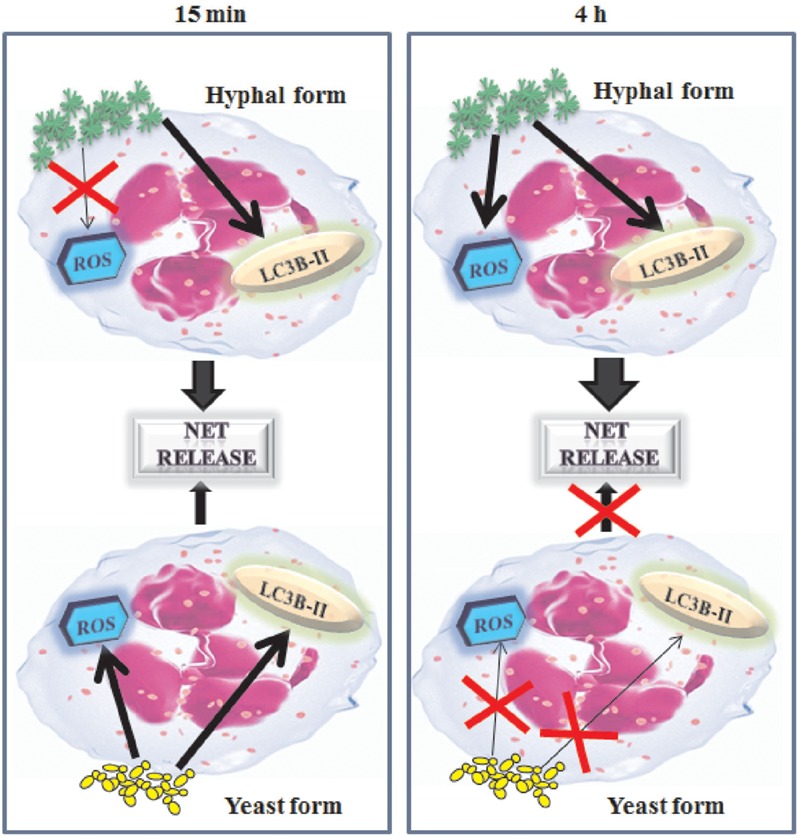
**Schematic representation of NET’s mechanism formation in response to *C. albicans*.** *C. albicans* hyphae induces rapid NET via autophagy (LC3B-II) and *C. albicans* yeast both via autophagy than reactive oxygen species (ROS). Later, after 4 h of contact with PMNs, only hyphal form induces NET formation by using autophagy as well as reactive oxygen species.

It has been reported that an inflammatory components is a key factor for NET release including the activation of transcription factor NF-κB (Lapponi et al., 2013). Furthermore NF-κB activation has been involved in neutrophil interaction with *C. albicans* (Giraldo et al., 2010). Indeed, NF-κB activation is observed in neutrophils treated with all fungal cells tested, including the inactivated fungal cells for both at 15 min and 4 h of stimulation. In this study, we demonstrate, for the first time, that the involvement of NF-κB activation, in NETs release, occurs in response to hyphal cells form consistently at 15 min and 4 h and in response to live and inactivated cells, only, after 4 h of incubation. A scheme of the principal mechanism involved in *C. albicans* NET-induced is reported in **Figure 8**.

Neutrophil extracellular traps is suggested as a mechanism to capture and kill *Candida* hyphae and yeast (Urban et al., 2006). In our experimental system, only a low percentage of CALY was killed by NETs, conversely NETs showed a remarkable killing activity toward CAH. Indeed, accordingly to the capacity of neutrophils to discriminate between yeasts and filaments of *C. albicans*, the study from Wozniok et al. (2008) reports that human neutrophils are differently activated in response to distinct *C. albicans* morphotypes. The filamentous form is able to induce ERK signaling that promotes motility of neutrophils and killing of *C. albicans* filaments. Conversely *C. albicans* yeast is unable to induce ERK and killing via motility (Wozniok et al., 2008). Given that only a negligible percentage of *Candida* yeast is killed by NET, and that NET is generated in response to inactivated yeast, it is conceivable that NET is released not, only, to kill microorganisms, but, also, to prevent dissemination of microorganisms by trapping them or for other unknown function. This is consistent with a recent paper demonstrating that, indeed, the trapped microorganisms could be alive (Menegazzi et al., 2012).

Collectively our data show that, both *C. albicans* morphotypes induce rapid NETs formation. In a early phase (15 min) autophagy plays an important role in inducing NETs in response to hyphae and collaborate with ROS in inducing NET in response to yeast. Lately (4 h) autophagy and ROS are involved in NETs release in response to hyphae, while, at this time NETs is undetectable in response to yeast and an inducible soluble factor could be involved in this phenomenon. Importantly, NET is implicated in hyphal killing, but retains a marginal role in destroying yeast cells. These results provide evidence that the *C. albicans*, yeast and hyphae, drive distinct kinetics and mechanisms of NET induction and suggest that the inhibition of NET by live yeast cells, in a 4 h, could be due to a release of soluble factor generated during *Candida*-neutrophils contact.

## Author Contributions

SK: Designed, performed experiments, and analyzed data. SP: designed experiments and analyzed data. PM: designed and performed experiments. AV: designed experiments, analyzed data, and wrote the paper. CM: designed experiments, analyzed data, and wrote the paper.

## Conflict of Interest Statement

The authors declare that the research was conducted in the absence of any commercial or financial relationships that could be construed as a potential conflict of interest.
